# Deep Learning for Image Enhancement and Correction in Magnetic Resonance Imaging—State-of-the-Art and Challenges

**DOI:** 10.1007/s10278-022-00721-9

**Published:** 2022-11-02

**Authors:** Zhaolin Chen, Kamlesh Pawar, Mevan Ekanayake, Cameron Pain, Shenjun Zhong, Gary F. Egan

**Affiliations:** 1 Monash Biomedical Imaging, Monash University, Melbourne, VIC 3168 Australia; 2 Department of Data Science and AI, Monash University, Melbourne, VIC Australia; 3 Department of Electrical and Computer Systems Engineering, Monash University, Melbourne, VIC Australia; 4 Turner Institute for Brain and Mental Health, Monash University, Melbourne, VIC Australia; 5 National Imaging Facility, Brisbane, QLD Australia

**Keywords:** Magnetic resonance imaging, Post-processing, Image enhancement, Artefact correction, Noise, Super-resolution

## Abstract

Magnetic resonance imaging (MRI) provides excellent soft-tissue contrast for clinical diagnoses and research which underpin many recent breakthroughs in medicine and biology. The post-processing of reconstructed MR images is often automated for incorporation into MRI scanners by the manufacturers and increasingly plays a critical role in the final image quality for clinical reporting and interpretation. For image enhancement and correction, the post-processing steps include noise reduction, image artefact correction, and image resolution improvements. With the recent success of deep learning in many research fields, there is great potential to apply deep learning for MR image enhancement, and recent publications have demonstrated promising results. Motivated by the rapidly growing literature in this area, in this review paper, we provide a comprehensive overview of deep learning-based methods for post-processing MR images to enhance image quality and correct image artefacts. We aim to provide researchers in MRI or other research fields, including computer vision and image processing, a literature survey of deep learning approaches for MR image enhancement. We discuss the current limitations of the application of artificial intelligence in MRI and highlight possible directions for future developments. In the era of deep learning, we highlight the importance of a critical appraisal of the explanatory information provided and the generalizability of deep learning algorithms in medical imaging.

## Introduction

Magnetic resonance imaging (MRI) is a non-invasive in vivo biomedical imaging modality that underpins many recent breakthroughs in biology and medicine. Compared with other imaging modalities, MRI is superior in providing excellent soft-tissue contrast. MRI can be applied to a diverse range of clinical and research applications to visualize anatomical structures, measure biophysical functions and metabolism, as well as quantify perfusion and diffusion weighted microstructures in soft tissues and organs.

With the ever-increasing demand for shorter imaging time and higher image resolution, MRI increasingly suffers from low signal to noise ratio (SNR) and is prone to image artefacts arising from subject motion and image distortion. These pose crucial challenges to accurately and efficiently post-process MR images. Conventional image enhancement and artefact correction techniques have proven to be useful for improving image quality in MRI including denoising [1], geometric distortion correction [2], and correction of subject movement [3]. With the advent of artificial intelligence and machine learning, especially deep learning algorithms, there is great potential to further improve image quality in MRI, and many early works have demonstrated significant gains in image quality.

**Fig. 1:**
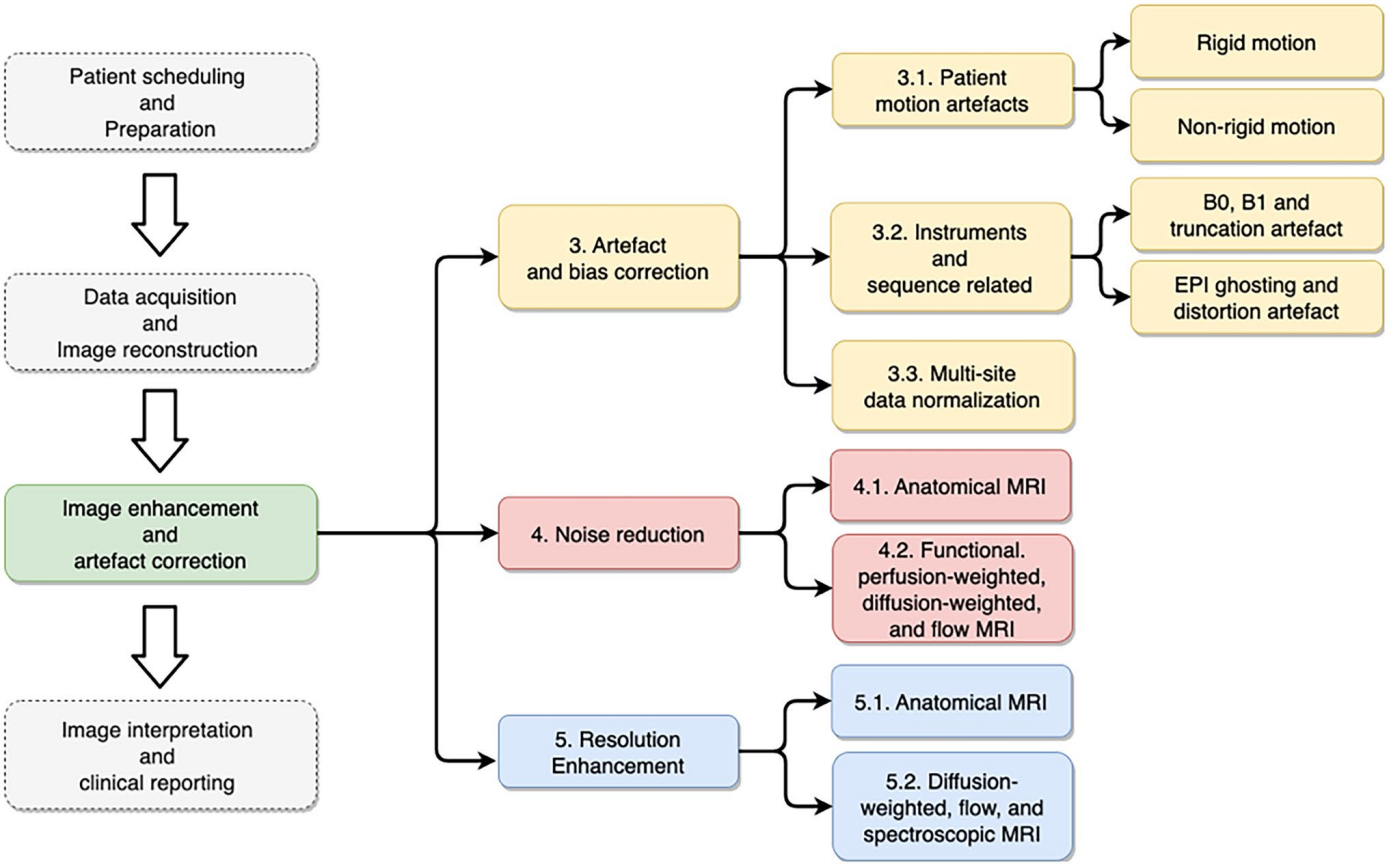
Overview of the scope of the review paper which focuses on the post-processing steps after image reconstruction and includes MRI artefact correction, noise reduction, and resolution enhancement

Deep learning has proven to be useful in various steps of the clinical imaging workflow including patient scheduling, data acquisition and reconstruction, image enhancement and correction, and interpretation of results (see Fig. 1). Significant improvements in workflow efficiency, data quality, and interpretation efficiency have been reported [4]. For example, in the image reconstruction literature, several papers have comprehensively reviewed the image reconstruction algorithms using deep models for improved image reconstruction accuracy [5, 6]. For accurate and robust image interpretation, Cai and colleagues reviewed deep learning for image classification and segmentation tasks [7]. Furthermore, McBee et al. have provided an overview of deep learning in radiology practice covering topics including disease detection, classification, segmentation, and quantification [8]. In this review, we particularly focus on post-processing algorithms for image quality enhancement and artefact correction, as many existing research works have demonstrated that deep learning models are well suited for image post-processing tasks in MRI.

In this paper, we will first provide an overview of the recent development of deep learning for MRI post-processing including (i) image artefact reduction, (ii) denoising for different MRI contrasts, and (iii) improvement for image resolution. We aim to provide researchers with an overview of deep learning approaches for post-processing MR images and discuss future perspectives in these applications.

## Overview of Deep Learning Models in MRI Post-Processing

Many MR post-processing tasks can be formulated as image-to-image transformation problems where deep learning models are used to capture the nonlinear relationships between image inputs and outputs. In both rigid and non-rigid motion correction tasks, motion-corrupted images are fed into deep neural networks (DNNs) which produce the corresponding motion-free images. To correct other artefacts, such as Gibbs ringing, image bias due to B0 inhomogeneity, ghosting, and distortion artefacts, DNNs take images with artefacts as the inputs and output artefact-free images. Similarly, various neural networks are implemented to reduce noise or enhance image resolution in MRI images with supervised or unsupervised training.

### Network Architectures

Among various types of deep neural networks, convolutional neural networks (CNNs) are the most commonly used neural network architecture in medical image processing [9]. The convolution operations or kernels are the major building blocks of any deep CNN architecture, which are specialized linear operations that are capable of detecting translation invariant features from images, including 1D, 2D, and 3D Conv layers, while several works use long short-term memory (LSTM) and multi-level perception (MLP), as illustrated in Table 1.

**Table 1:** Summary information of various MRI post-processing tasks

Task	Motion correction	Instrumental artefacts correction	Sequence artefacts correction	Multi-site normalization	Noise reduction	Noise reduction	Super-resolution
Scopes	Rigid motion, non-rigid motion	Gibbs ringing, B0 inhomogeneity	EPI ghosting and distortion	Multi-site normalization	Anatomical	fMRI, DWI, perfusion, ASL	Anatomical, DWI, spectroscopic
Encoder types	2D Conv	2D Conv, 3D Conv	2D Conv, 3D Conv	2D Conv, 3D Conv	2D Conv, 3D Conv	1D Conv, 2D Conv, 3D Conv, LSTM, MLP	2D Conv, 3D Conv, MLP
Model types	CNN, Inception, ResNet, FCN, VAE, U-Net	CNN, U-Net, ResNet50	AE, U-Net	CNN, U-Net, CNN with attention, VAE, ResNet	CNN, AE	CNN, U-Net, FCNN, transformer	CNN, U-Net, AE
Training types	Fully supervised, adversarial	Fully supervised, adversarial	Fully supervised, adversarial	Fully supervised, adversarial	Fully supervised, adversarial	Fully supervised, adversarial	Fully supervised, adversarial
Loss	MAE, MSE, adversarial loss	MAE, MSE, adversarial loss	MAE, MSE, SSIM loss, gradient loss	MAE, perceptual loss	MAE, MSE, perceptual loss	MAE, MSE, SSIM loss	MAE, MSE, adversarial loss, SSIM loss. triplet loss
Input types	2D image, 2D patch, 3D patch	2D image	2D image, 2D patch	2D image	2D image, 2D patch	2D image, 3D image, fMRI time series, diffusion-weighted data	2D image, 2D patch, 3D patch
Inputs	Images/patches with simulated motion artefacts	Images/patches with simulated Gibbs artefacts or simulated phase errors	Images/patches with ghosting and distortion	Images acquired on different scanners or with different parameters	Images/patches with simulated Gaussian/Poisson/Rician noise	Noisy images and fMRI, undersampled DWI data	Low-resolution images
Outputs	Motion-free images/patches	Artefact-free images/patches	Artefact-free images/patches	Labels for the task of interest	Noise-free image/patch	Noise-free images and fMRI, fully sampled DWI data	High-resolution images
Training data source	Motion artefacts are simulated by adding phase error in k-space	Gibbs ringing artefacts are simulated with cropped kspace, created bias fields for simulating B0 inhomogeneity	Artefact-free images are generated by post-processing steps	Multi-site data acquisitions	Simulation by adding noises to the clean image to generate noisy training samples	Noise-free data is generated by applying post-processing steps	Simulation by undersampling k-space to create low-resolution training samples
Anatomical regions	Brain, liver, abdomen, pelvis	Brain, knee, respiration	Brain	Brain	Brain, knee	Brain, prostate, whole body	Brain, prostate, knee, fetal, cardiac, torso
Metrics	SSIM, RMSE, MMI	SSIM, PSNR, RMSE, HFEN, GSR	GSR, MSE, Mutual Information	MAE, DICE,	SSIM, PSNR, IFC	SSIM, MAE, MSE, RMSE, PSNR	SSIM, PSNR, RMSE, DICE
Examples	MocoNet [19], MedGan [17], NAMER [49]	GRA-CNN [50], InHomoNet [51], DeepResp [34]	Synb0-DisCo [52], S-Net [53]	AD2A [54], DeepHarmony [47]	dDLR [55], RED-WGAN [48], DABN [56]	DeNN [31], 3DConv-LSTM [57], DNIF [24], STFNet [58]	DeepVolume [45], SRGAN [59], SR-q-DL [46]

**Fig. 2:**
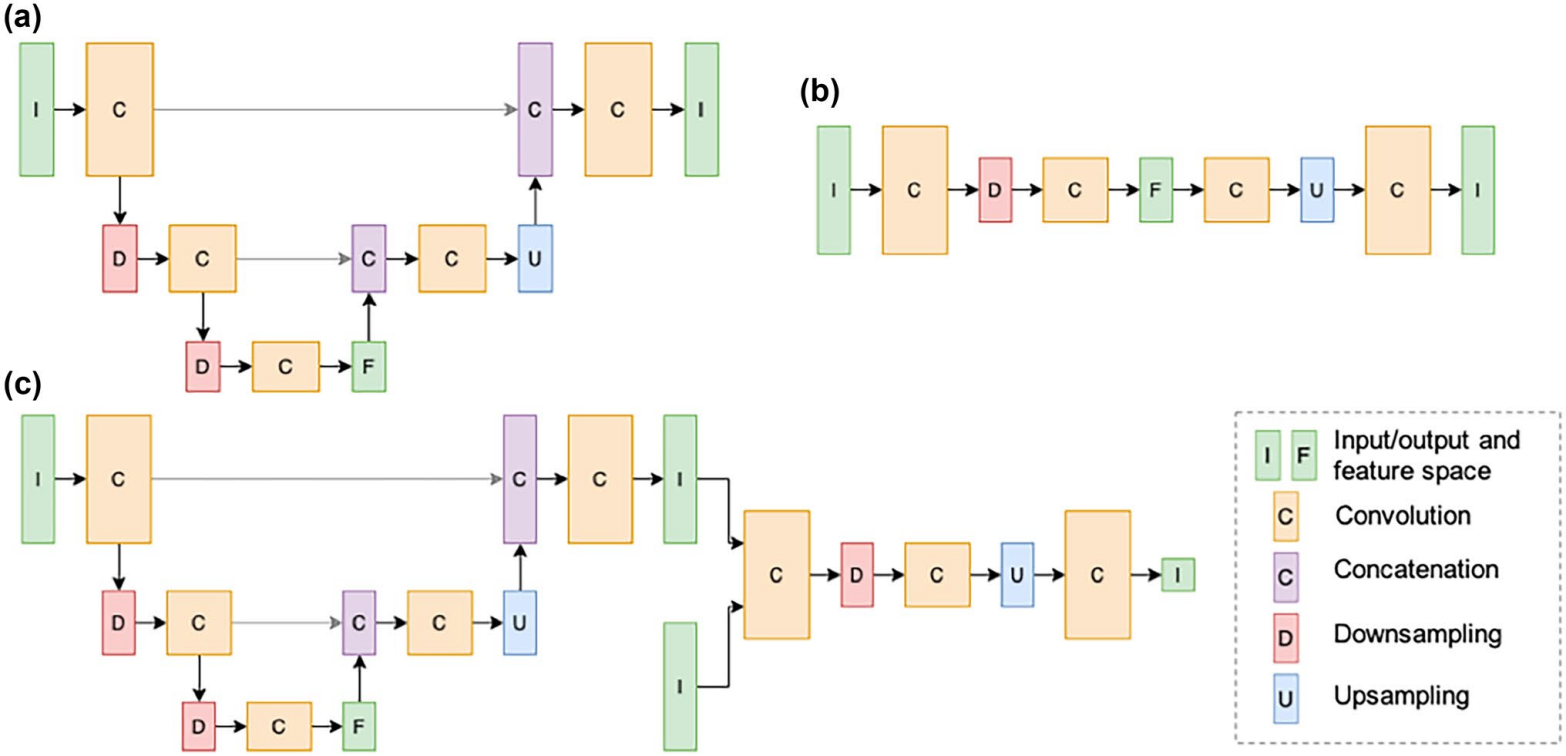
(**a**) Unet consists of a fully convolutional encoder and decoder interconnected by concatenating feature maps which assists in propagating spatial information to deep network layers. (**b**) Autoencoder consists of an encoder which maps images to a latent space of reduced dimensionality and a decoder which maps the latent space vector to image space. The dimensionality reduction mitigates random variations in the input while preserving image features necessary for image reconstruction. (**c**) Generative adversarial network consists of a generator network which produces an estimate of a ground-truth image and a discriminator which attempts to discern between synthesized images and ground truth images. Parameters for each network are updated in an alternating fashion resulting in generator outputs which are indistinguishable from ground truth images from the perspective of the discriminator.

CNN architectures, such as U-Net and its extensions, are the widely used models for MR image quality enhancement and artefact correction. Table 1 includes the model architecture types covered in this survey and shows that U-Net is one of the most popular network architectures for mapping from source images to target images, among a variety of customized CNN networks. The 2D U-Net model was first designed for image segmentation tasks [10] as a fully convolutional neural network (FCN) architecture (Fig. 2a). It consists of an encoder that contracts spatial information to latent feature maps with convolution operations and a decoder that expansively performs up-sampling step propagating spatial information from the input to deep layers of the network, in order to preserve spatial features from the input image that are relevant to the output image. Since the first introduction of the U-Net model, variations to the initial architecture to handle fully 3D data [11–13] have also been implemented (Table 1).

Similar to U-Net architecture, some works applied autoencoders (AE) with residual connections to enhance medical images [14, 15]. As shown in Fig. 2b, an autoencoder architecture is generally the composition of two parts: an encoder which maps an input image to latent space features in lower dimensions and a decoder which predicts an output from the latent space. Dimensionality reduction forces the neural network to give priority to learning those features which most significantly contribute to minimizing the loss, thus reducing undesirable features such as noise and artefacts [16]. Variational autoencoder (VAE) was applied for applications including correcting motion artefacts [17] and normalizing multi-site image data [18]. With the recent success in self-attention-based transformers, there have been several attempts to study the effectiveness of these networks on MRI post-processing tasks, especially on noise reduction. The transformers consist of fully connected layers and operate on image patch embedding in order to exploit long-range dependencies between visual MR features.

### Network Training

Training a neural network is the process of optimizing its parameters to minimize the loss between the predicted and target images or patches. Loss functions determine which image characteristics will be used by the neural network to match with the ground truth data. In the scope of MR image enhancement and artefact correction, the mean absolute error (MAE) using L1 norm and mean square error (MSE) using L2 norm are the two most widely used to compute the pixel-wise residual component between neural network outputs and ground truth images or patches [13, 19–21]. Some works incorporated multiple losses, e.g., MAE or MSE, and Structural Similarity Index (SSIM) to improve perceptual quality of output images [22–24], where the SSIM loss measured the similarity of the output and target images [25]. Different from the above hand-crafted loss functions, perceptual loss was proposed to measure the high-level perceptual and semantic between the neural network generated images and target image in feature space, instead of computing the pixel differences [26]. It was originally designed for image style transfer problems, later applied to MRI image processing with pre-trained VGG networks as the loss network, such as motion correction [17], contrast synthesis [27], and noise reduction [28].

In the context of MRI image quality enhancement, the process of optimizing model parameters is mostly formed as a fully supervised or adversarial training framework. In a fully supervised setup, the models are trained with explicitly defined pairs of input and output. For example, to correct artefacts, models are optimized by using the loss functions between network outputs and artefact-free ground truth images or patches as the supervisory signals, through the process of gradient descent. The fully supervised learning framework was widely used for motion correction [19, 29], noise reduction [30–32], instrumental, and sequence artefact correction [33–36]. For super-resolution tasks, similar setups were adopted, where the target images or patches were in high resolution and the inputs were low-resolution counterparts.

In contrast to fully supervised learning frameworks, some methods applied unsupervised learning techniques to train neural network models without explicitly labelled training pairs. Among those, generative models such as the generative adversarial network (GAN) framework, as shown in Fig. 2c, are popular techniques. GAN operates two networks, a generator and a discriminator, which are updated in an alternating fashion and converged to a state where the generator output is indistinguishable from the ground truth images according to the discriminator. GANs have demonstrated good performance in natural image processing tasks such as image synthesis [37, 38] and image enhancement [39, 40]. Other established methods include AE, VAE, or using self-supervised learning algorithms which have also been applied for MRI post-processing tasks. Furthermore, some works utilize the mutual information between multi-modality contrasts for rigid motion correction where a neural network is trained in an unsupervised way to align T1, T2, and FLAIR images by minimizing the normalized cross-correlation measurement [79] and correct susceptibility artefacts in images acquired with echo planar imaging (EPI) sequence with pairs of reversed phase-encoding images without the need of artefact-free images [53]. Similarly, pseudo labels can also be simulated to form as a self-supervision framework [144, 166]. Moreover, instead of being formulated as learning problems, deep image prior (DIP)-based methods use CNNs as a regularizer and form as an optimization problem at inference time [190]. In MRI, DIP has been successfully applied to image denoising of structural images [103] and diffusion-weighted images [130].

### Performance Evaluation

To quantitatively evaluate the model performance, multiple metrics are used to measure the similarity between the enhanced images by the DNNs and the ground-truth reference images. Mean squared error (MSE) and root mean square error (RMSE) measures the pixel-wise difference between the artefact-free reference images and the artefact-corrected network output images [18, 24, 41] or between the high-resolution ground-truth images and the synthesized super-resolution images [42, 43], where lower values indicate better model performance. Similarly, peak signal-to-noise ratio (PSNR) is another objective metric which is inversely proportional to the logarithm of the pixel-wise MSE between the generated image and the reference image. SSIM is a widely used metric for the level of similarity between images, especially image edges. PSNR and SSIM are the two most widely used metrics in validating MRI image quality enhancement models [20, 44–46]. Besides, high frequency error norm (HFEN) [33], dice coefficients [12, 47], percent volume difference (PVD) [47], and ghost-to-signal ratio (GSR) [34, 35] are also used by some researchers, as well as mutual information-based metrics, including information fidelity criterion (IFC) [48] and normalized mutual information (NMI) [17].

## MR Image Artefact and Bias Correction

Artefacts in MR images emerge from different sources such as human physiology and instruments. In this section, we have categorized common MR imaging artefacts as shown in Fig. 1.

### Patient Motion Artefact

Patient motion during MRI acquisition frequently occurs due to either involuntary motion of the patient or physiologically related tissue movements. Motion during the scan has been a long-lasting issue in MR imaging [49, 50] and can manifest as image artefacts including ringing, ghosting, blurring, or a combination thereof [49, 50]. The type of image artefact depends on both the severity of the motion as well as the time point at which motion occurred during the scan [19]. For instance, motion occurring during the acquisition of high-frequency components will manifest as ringing and blurring, while motion during the acquisition of low-frequency components will result in ghosting. The two broad approaches to correct motion artefacts apply either (i) prospective or (ii) retrospective motion correction methods. Prospective motion correction [51–55] involves detection of patient motion in real-time and modification of the scanner gradients to maintain the relative position of the patient in the field of view (FOV). In principle, prospective motion correction is an efficient method to correct motion artefacts as it can compensate for a patient’s motion at the source. However, in practice, it is difficult to accurately estimate a patient’s motion in real time. Retrospective motion correction [56–58] methods do not compensate for patient motion at source but instead correct the image artefacts that result from the motion.

Motion types can be separated into rigid motion (e.g., head motion) and non-rigid motion (e.g., whole-body motion).

#### Rigid Motion Correction

The use of deep learning to correct motion artefacts was first explored by Sood et al. [59], Godenschweger et al. [60] and Zaitsev [61]. Several methods [19, 23, 29, 41, 62–65] used simulated motion artefacts to generate paired datasets for training DL models. Pawar et al. [19] and Sood et al. [59] simulated motion artefact for 3D MPRAGE images using randomly generated motion parameters with six degrees of freedom and trained an encoder-decoder Unet to map motion-corrupted images to the motion-free images. The authors compared the DL motion correction results with a method based on the entropy minimization [66] technique and demonstrated that the DL-trained model had superior performance in comparison to the entropy motion correction method. Godenschweger et al. [60] simulated motion for the 2D images using a patch-based CNN approach to remove artefact from each small patch of image and assembled the patches to generate a motion-corrected image. Gallichan et al. [67] used an end-to-end training to generate a full 2D image. Recently, Ghodrati et al. [68] used a conditional generative adversarial network which uses 3D patches instead of 2D patches and demonstrated that the use of 3D patches improves the motion correction accuracy compared to using 2D slices. In a separate work, Johnson et al. [69] proposed MC2-Net, which uses multi-contrast T1, T2, and flair images simultaneously to first align the T1, T1, and flair images using an unsupervised alignment DL network and subsequently process the aligned multi-contrast images through a separate DL motion correction encoder-decoder network. They demonstrated that using multi-contrast MR images improves the reconstruction quality of the images compared to using a single contrast image.

Motion simulation is an important aspect to develop DL motion correction models. Pawar et al. [70] developed a motion simulator that generates realistic motion trajectories and corresponding motion corrupted images. Although acquiring in vivo pair training data is a complex experimental task, a study by Küstner et al. [17] attempted to use the paired data from 18 subjects, one acquired with the subject moving and another with the subject still. They used the motion paired data to compare two different motion correction DL models including autoencoders (AE) and generative adversarial networks (GAN) for head, abdomen, and pelvis imaging.

Bilgic et al. and Brown et al. [41, 62] further developed the DL motion correction model to integrate data consistency during the motion correction step to ensure that the motion-corrected image was consistent with the acquired data. Motion correction using iterative optimisation algorithms to estimate both motion parameters and motion-corrected images has been explored in [66] without the use of deep learning. However, a major limitation of methods that use iterative optimisation algorithms is their computational complexity and their failure to converge due to the large search space. Integration of DL models [71] in order to regularize the iterative methods has enabled minimization of the cost function in a reasonable computing time.

**Fig. 3:**
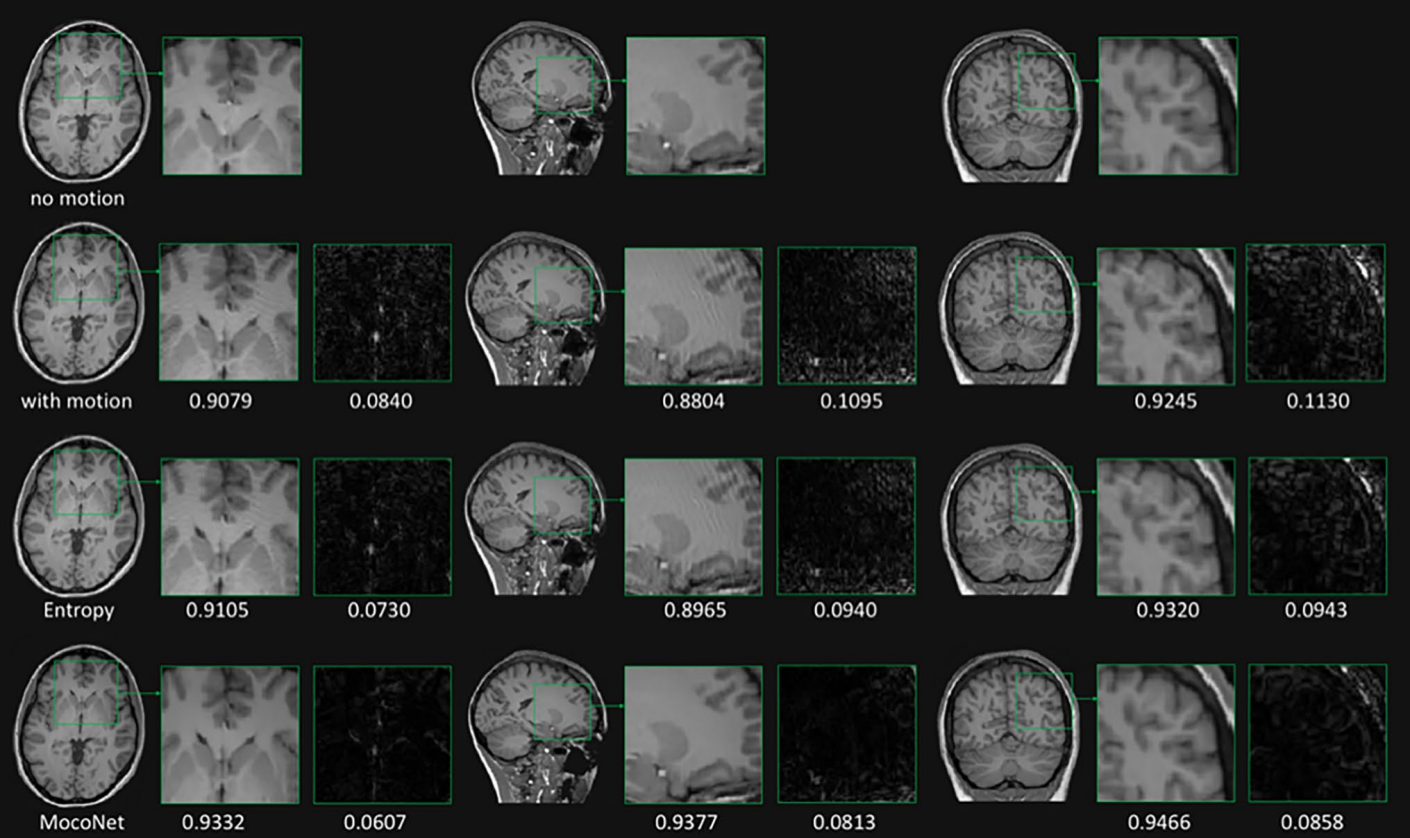
Motion degraded (left-hand column) and motion-corrected (right-hand column) images highlighting the image quality improvement for a case with a brain tumor. The ringing motion artefacts were removed from the images without degrading the diagnostic image quality [72]

Many works [19, 21, 23, 29, 31, 41, 49, 70–75] have demonstrated that motion can be corrected using DL approaches and simulated data, with some studies validating the proof of concept using few volunteer scans. However, the performance of these methods in the clinical setting has been largely unexplored. Figure 3 shows an example of the motion correction comparing two retrospective motion correction methods, one DL-based (MocoNet) and another iterative regularization-based entropy minimization method (taken from [72]). The authors demonstrated improved image quality after motion correction using MocoNet.

#### Non-rigid Motion Correction

Modelling non-rigid motion such as respiratory- or cardiac-related movements is more challenging than rigid motion. Tamada et al. [23] developed a motion correction model to correct for the respiratory artefacts in liver imaging. Although the task was to model non-rigid motion, they approximated chest movement as rigid motion based on the assumption that the chest moved in only one direction during breathing. They modelled periodic motion with random phase and frequency to simulate breathing and simulated random motion to simulate a break in the breath-hold. A patch-based approach was used to remove the motion artefact using a seven-layer CNN without any downsampling layers. In a separate study, Zhao et al. [57] approximated respiratory motion as rigid motion to generate respiratory motion corrupted cardiac cine data and then used an autoencoder network with adversarial training in an unsupervised manner.

#### Evaluation of Deep Learning-Based Motion Correction

Although most of the current literature focuses on the development of novel methods for motion correction in MRI, several methods have evaluated the effect of DL motion correction in clinical and research applications.

Two studies [29, 72] validated the effect of motion correction in the clinical setting. In both studies, a visual grading score (VGS) was assessed by multiple trained radiologists for the quality of images. Johnson et al. [72] performed 5-point VGS on brain images for nine neuroanatomical regions, while Kromrey et al. [29] performed a VGS study on the liver using a 4-point scale. They both concluded that the visual appearance of the motion-corrected images was improved compared to the motion-corrupted images. Pawar et al. also concluded that 13% of the repeated scans can be avoided and 90% of the motion degraded scans can be improved using motion correction.

To evaluate motion correction for segmentation tasks, Maclaren et al. [64] compared two approaches for motion correction, namely, (i) image motion corrected with a DL model and then used in a segmentation algorithm and (ii) motion-corrupted images included as training examples in a DL segmentation model. They demonstrated that the second approach that included motion corrupted images as training examples for DL segmentation models outperformed models that corrected images corrupted with motion artefacts. Johnson et al. [73] performed a similar study for neonatal brain segmentation and demonstrated that DL motion correction improved segmentation accuracy. Shaw et al. [74] demonstrated that DL motion correction improved cortical surface reconstruction of the brain and that quality control failures were reduced from 61 to 38 by the use of a DL motion correction algorithm in a Parkinson’s disease study of 617 patients.

Diffusion parameter estimation can be affected by misalignment of echo planar image (EPI) volumes. Conventionally, volumes with motion greater than a predefined threshold are discarded retrospectively to avoid bias in estimation of the diffusion parameters. Terpstra et al. [75] proposed a 3D patch-based CNN to reduce variability associated with residual EPI volume misalignment. The method used a separate database to train a 3D patch CNN for each subject during the correction stage and demonstrated that the method could reduce the variability in diffusion parameter estimation due to motion. Table 2 highlights the advantages and limitations for popular DL-based motion correction methods.

**Table 2:** Advantages and limitations for deep learning methods in MRI motion correction

Literature	Advantages	Limitations
Kustner et al. [17], Sommer et al. [71]	• Patch-based image to image translation networks and these methods are efficient in GPU memory and thus easier to implement in practice	• Motion artefact is of global appearance, and the patch-based methods may not be the optimal solution
Johnson et al. [73]	• A full 3D motion simulation and 3D DL network for motion correction	• 3D Unet is used, but the field of view coverage in slice direction is only limited to 8 slices
Lee et al. [79]	• Using information from multi-contrast images to correct motion artefact	• Multiple contrasts may not be available for all the studies, and the method is prone to image registration errors across multiple contrasts
Pawar et al. [19, 19]	• Full 3D motion simulation, large dataset for training, validation on simulated, as well as real motion degraded images in clinical setting	• Processes only 2D slices that may result in slice to slice variations when viewed from the other orthogonal plane
Bilgic et al. [41], Haskell et. al. [49]	• Provided estimates for both motion parameters and motion corrected images	• A two-step approach where DL is used as a preprocessing step for an iterative motion correction model, and potentially multiple sources of errors may add together
Ghodrati et al. [68], Terpstra et al. [75], Tamada et al. [23]	• Methods developed for dynamic MR imaging and can correct for non-rigid motion artefact including cardiac cine and DCE liver MRI	• Small dataset used for training with proof of concept validation with limited clinical evaluation
Khalili et al. [83], Duffy et al. [84], Gong et al. [85], Shaw et al. [74]	• Focused on practical application of motion correction methods by assessing the downstream tasks such as image segmentation, cortical surface reconstruction, and diffusion parameter estimation on motion corrected images	• Actual motion corrected images are not compared with ground truth images

### Instrument and Pulse Sequence-Related Artefacts

A major source of artefacts in MR images stem from the choice of acquisition parameters at the MR hardware operational limit, including at high slew rates and high gradients, and because of the non-uniform B0 and B1 fields. In this section, we categories artefacts based on the hardware limitations, choice of acquisition sequence, sequence parameters, and scanner non-uniformity.

#### B0, B1, and Truncation Artefact Correction

Truncation of the high-frequency k-space data is frequently employed to reduce scan time and often results in Gibbs ringing artefact. Loktyushin et al. [76] and Wang et al. [33] proposed a deep learning-based network trained on simulated Gibbs ringing data to remove such artefacts. The model consisted of a four-layer CNN to reduce ringing artefact in the image domain and ensure data consistency of the acquired low-frequency components that successfully reduced the ringing artefact for T1 and DWI images. Wang et al. [33] developed a small CNN similar to [76] but without data consistency for the acquired k-space points. Separately, Muckley et al. [20] developed a CNN model-based solely on non-MR images (ImageNet natural image dataset) to remove Gibbs ringing arising from partial Fourier imaging which demonstrated that ringing artefacts can be effectively removed using a model trained on different input images.

Magnetic field B0 inhomogeneity results in varying Larmor frequency spatially and thus the artefacts. B0 inhomogeneities can produce image biases, blurring, shading, curved slice profiles, and banding artefacts. B0 inhomogeneity artefacts are more pronounced in gradient echo and echo planar imaging sequences due to lack of refocusing RF pulses and long read out. Another aspect of B0 inhomogeneity is poor fat saturation since the difference between fat and water frequency is not fixed in the presence of B0 inhomogeneity. Non-uniform intensity biases can also be caused by improper coupling of the receiver coils and non-uniform B1 due to inhomogeneity of the radiofrequency excitation and receive coils. Sommer et al. [77] proposed a generative adversarial network with a 3D pixel and histogram loss function to remove non-uniform intensity (bias field) from MR images. They trained the network on simulated brain datasets and tested the algorithm on real brain and abdomen images. Respiratory motion can also introduce B0 inhomogeneity artefacts in T2*-weighted images due to thoracic cage movement. An et al. [34] proposed DeepResp to remove respiratory-induced B0 inhomogeneity artefacts that used artefact-free complex-valued images and respiratory motion curves to simulate phase errors in k-space. A DL network was trained to estimate and remove the phase errors in the phase encoding direction from corrupted k-space data to recover artefact-free images.

#### EPI Ghosting and Distortion Artefacts

EPI is a fast-imaging sequence that acquires the whole slice in one excitation by traversing the full k-space in a predefined manner. For a rasterized zig-zag trajectory, each consecutive phase encode (PE) is acquired by traversing k-space in the opposite frequency encoding direction. This can result in two major artefacts in EPI images: (i) ghosting in the reconstructed images due to eddy currents generated during the reversal of frequency encoding direction and (ii) phase errors arising from slight B0 inhomogeneities that accumulate during the readout time and cause geometric distortions in the reconstructed images.

**Fig. 4:**
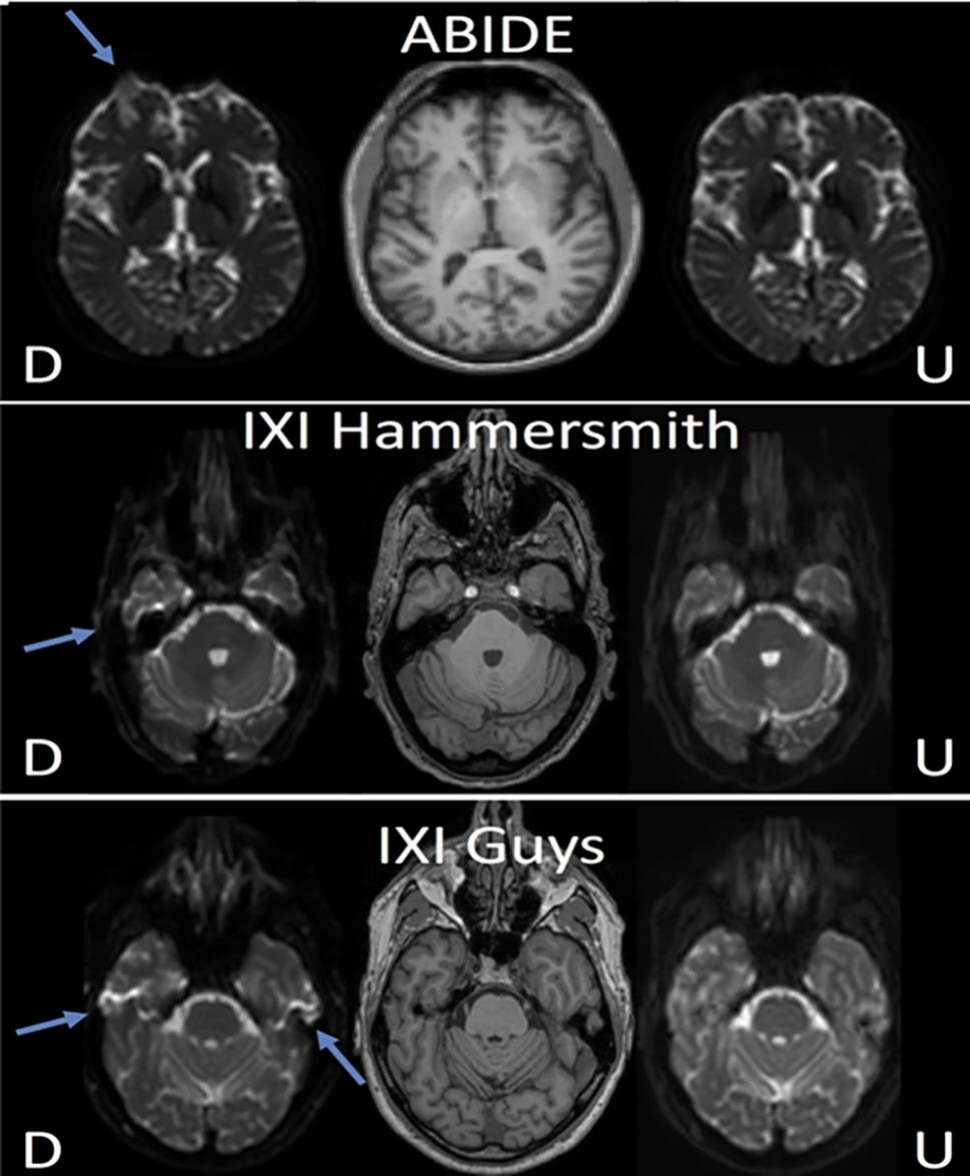
EPI distortion artefact correction in [80] form three datasets. D: distorted image, U: undistorted images formed using distorted and T1 weighted as input to a 3D Unet

Ghosting in the phase encoding direction can be corrected using two readouts in the opposite frequency encoding direction that calculate the phase errors due to the fast switching gradients. Lee et al. [35] proposed a deep learning-based method in the k-space domain to simultaneously correct for ghosting artefacts and reconstruct EPI images from undersampled data. In [36, 78–80] different DL approaches were proposed to correct EPI geometric distortions. Ghaffari et al. [78] proposed a 2D Unet approach that used a distorted B0 image along with a co-registered T1-weighted image to correct for distortion in the EPI images using only one EPI scan. They further improved the method in [80] and implemented a 3D Unet to correct for the EPI distortion (Fig. 4). Both methods were compared with the TOPUP method from the FSL toolkit [81, 82], a popular tool for distortion correction, and demonstrated similar performance in distortion correction without using an extra scan in the opposite direction. Separately, Hu et al. [36] proposed a 2D Unet for EPI distortion correction in the context of diffusion MRI. The method used distorted diffusion EPI images in seven diffusion directions and T2-weighted images as input to the patch-based 2D Unet to correct for EPI distortion in all seven EPI images simultaneously. Lee et al. [79] separately developed a DL method for EPI distortion correction with the aim of reducing the processing time compared to the conventional methods including TOPUP [81, 82] and TISAC for susceptibility distortion correction [83]. They used a 3D Unet to estimate the B0 distortion field map using an unsupervised approach, and the estimated distortion field maps were used to correct the EPI distortion. This approach still required EPI scans in two opposite PE directions that mimic the conventional approach to remove distortion artefacts to reduce processing time. They demonstrated a 369 × and 20 × processing speed increase in comparison to TOPUP and TISAC, respectively.

### Multi-site Data Normalization

Variations in scanner configuration and protocol make the analysis of data from different imaging sites highly challenging. Images from a subject scanned at two different sites can result in images with highly variable quality. Characterization and removal of such variability is of critical clinical and research importance that can be done using data normalization methods.

Grigorescu et al. [12] proposed a DL method for data normalization of T2-weighted images using an image domain adaptation network to harmonize the input T2-weighted image for gray/white matter and CSF segmentation. The model consisted of two sequential networks, the first being a normalization network which processed the input image to suppress scanner specific variation, followed by a segmentation network. The model was trained end-to-end with adversarial loss for the normalization network and dice loss for the segmentation network and was tested on neonatal brain segmentation data. Duffy et al. [84] proposed a similar idea on T1-weighted images and used a single network with adversarial loss to enforce learning of scanner independent features. Gong et al. [85] used adversarial loss to normalize data from different scanners and tested the CNN model for multiple tasks including segmentation (gray, white, CSF), regression (age prediction), and classification (gender prediction). Dewey et al. [47] proposed a Unet-based model for T1, T2, FLAIR, and proton density images to modify the contrast of the source image to a predefined target image contrast, with the target image used for further processing such as segmentation.

In [86], Tong et al. proposed a 3D image-to-image translation network to transform images from multiple sites to a reference site. The method required data from the same patient at different sites to train such a 3D model and demonstrated reduction in the inter-scanner variation of fractional anisotropy measures. Moyer et al. [18] developed an autoencoder DL method for normalization of diffusion MRI data that used an autoencoder to extract a latent space representation of the image that was independent of the scanner. The encoder part of the network estimated an unbiased scanner-independent representation of the input diffusion image, while the decoder used the latent representation and the scanner protocol representation as inputs to reconstruct the diffusion image. The advantage of the autoencoder is that it does not require data from the same subject to be acquired at multiple sites. The method allows normalization of diffusion images from one scanner to another using only the representation from the second scanner. Unlike [86], the method does not normalize the scans to a reference site but converts data from one site to another site using the latent representation and scanner representations.

Synthetic MRI is quantitative and free of vendor-specific characteristics which is an ideal form of normalization. In [27, 87], multiple dynamic multi-echo sequences (MDME) were used to acquire raw data and multiple contrasts including T1, T2, proton density, and T2-FLAIR images using MR physics-based modelling. T2-FLAIR images generated using the MDME method are often corrupted with hyperintensities in brain-CSF and granular hyperintensities in the CSF region of the brain. Ryu et al. [27] proposed a supervised DL network that used paired images acquired using MDME and conventional T2-FLAIR sequences. The paired images were first co-registered, contrast matched, and intensity normalized before being processed through the network. Separately, Jenkinson et al. [87] proposed a GAN network that used multi-channel raw data as an input to the generator network and a discriminator to differentiate between true and fake T2-FLAIR images. They used adversarial training to obtain a generator that could generate realistic T2-FLAIR images from the raw data.

## Noise Reduction in MRI

Image noise is a long-standing issue in MRI. Improving SNR is increasingly challenging because of accelerated data acquisition and ill-posed image reconstruction methods. Noise in MR magnitude images is Rician distributed [88]. For accelerated data acquisitions, image noise can be spatially dependent on the corresponding reconstruction algorithms used, e.g., parallel imaging and compressed sensing. This poses particular difficulties for post-processing techniques to successfully denoise MR images.

Conventional post-processing-based denoising methods are classified into filtering methods, transform domain methods, and statistical methods (for detailed reviews, refer to [89, 90]). While spatial smoothing filters can be effective to remove additive noise, they often blur images especially small structures. Edge-preserving filters can mitigate image smoothing to a certain degree, and nonlinear filters such as anisotropic diffusion filters [91, 92] are useful to preserve anatomical details. Anatomical boundaries can also be preserved with non-local mean filters [93, 94] to exploit non-local image intensity and structural information while estimating and removing noise. The Block Matching 3D Filtering (BM3D) method applied a sparse representation for noise removal in a transformed domain [95, 96]. Awate et al. applied a nonparametric empirical Bayesian approach for Rician noise modelling and removal in MR images [97].

From the generic image processing perspective, CNNs have been very effective in the reduction of image noise (see [44] for an overview) with many MR image denoising techniques influenced by standard CNN models and variants. Deep learning denoising techniques for MR image post-processing have particularly focused on anatomical MRI applications, especially the brain, due to the fine anatomical details. There has also been a considerable amount of research on functional, perfusion-weighted, diffusion-weighted, and flow MRI.

### Noise in Anatomical MRI

Many studies have focused on noise removal in brain images due to the fine anatomical detail and high resolution of brain MR images. Most of these studies utilize CNNs and its variants for denoising. Several studies have incorporated GANs to learn the distributions of denoised MR images using the inherent generator-discriminator setup. With the recent invasion of transformers in computer vision, there have been several studies incorporating self-attention-based methods for denoising. Moreover, one of the recent focuses has been the quality enhancement of low-field anatomical MRI due to its innate advantages in accessibility and the higher vulnerability to noise.

Kidoh and colleagues reported an experimental study of brain MRI scans from five healthy volunteers that compared performance between three deep learning-based denoising methods, namely, denoising convolutional neural network (DnCNN), a shrinkage convolutional neural network (SCNN), and deep learning-based reduction (dDLR) [98]. The performance of dDLR was higher compared to DnCNN and SCNN with respect to Peak Signal to Noise Ratio (PSNR) and Structural Similarity Index (SSIM), and the image quality of dDLR was also superior to DnCNN and SCNN. Several groups have improved MR image denoising using deep image priors [99–101] and demonstrated improved performance compared with conventional filtering methods. Aetesam and colleagues proposed a deep CNN to remove Gaussian noise from brain MR images [30]. The method was inspired by the maximum a posteriori (MAP) with Gaussian noise and deep residual learning. Chauhan and colleagues combined a fuzzy logic approach with a CNN autoencoder to denoise brain MR images that showed improved performance compared to stand-alone fuzzy logic methods [14]. Apart from these, a ten-layer CNN [102], multi-channel residual learning CNN [103], CNN-DMRI [104], HydraNet [105], NNDnet [106], CMGDNet [107], 3D-Parallel-RicianNet [108], and a patch-based CNN [109] have been developed for accurate MRI denoising. Several other recent works incorporated CNN-based solutions for brain MRI denoising [110–115].

**Fig. 5:**
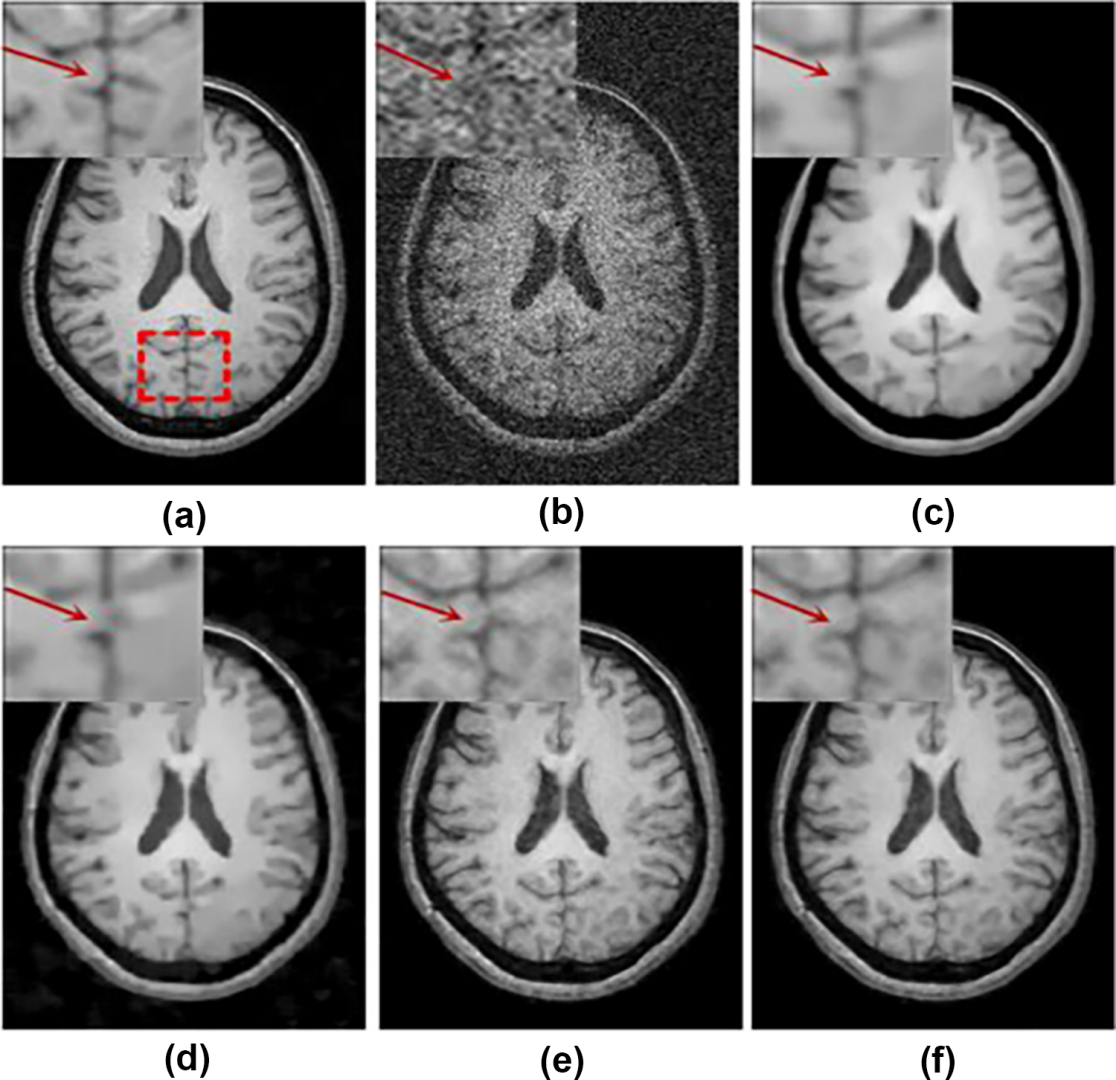
Comparison of image denoising on a T1-weighted image [30]: (**a**) noise-free image, (**b**) noisy image, (**c**) BM4D, (**d**) PRI-NLM3D, (**e**) CNN3D, and (**f**) RED-WGAN [48]

Furthermore, several works reported the incorporation of peripheral deep learning concepts including adversarial training and transfer learning to overcome noise in brain MRI. Ran et al. used a residual encoder–decoder Wasserstein GAN for simultaneous improvement of noise suppression and preservation of anatomical details [48] and compared deep learning-based and conventional methods (Fig. 5). Tian et al. introduced a novel MRI image denoising method using a conditional Generative Adversarial Networks (GAN) where a CNN is used as the discriminator network [116]. The model was trained by an adversarial loss function and tested on synthetic T1-weighted brain MR images with 10% noise level and outperformed several other methods in terms of denoising level and preservation of the anatomical structures. Many CNN-based methods for brain MRI denoising use squared Euclidean distance for training that produce overly smoothed output images. As an alternative, Panda et al. [28] introduced a perpetual loss which promoted restoration of visually desirable brain image features. This method surpassed the previous methods for the reduction of Rician noise.

Several groups have applied attention-based mechanisms and leveraged long-range dependencies to improve brain MR image denoising. Inspired by the attention-guided CNN networks, Hong et al. proposed a model to incorporate feature fusion and attention mechanisms to separate noise from observed MRI images [117] and produced competitive results. To overcome the limitations of conventional convolution and local attention in MR/PET denoising, Yang et al. [118] proposed a self-attention-based transformer model called the spatial-adaptive and transformer fusion network (STFNet). The STFNet utilizes a Siamese encoder to promote extraction of more relative and long-range contextual features and a transformer fusion encoder to establish local/global dependencies between high-level visual embedding of PET and MRI. Li et al. developed a progressively distribution-based neural network [119]. Unlike the conventional MRI denoising methods which utilize the spatial information around image patches, the method learned the pixel-level distribution information in a supervised manner. Through a series of experiments on synthetic, complex-valued and clinical MR brain images, the authors showed that the approach improved quantitative measures including PSNR and SSIM, as well as visual inspection of edge-like details and anatomical structures. Xu et al. aimed to simultaneously address long-range and hierarchical information and utilize similarity in 3D brain MR images for denoising [120]. They proposed a deep adaptive blending network (DABN) characterized by large receptive field residual dense blocks and an adaptive blending method. The overall results showed superior performance of DABN over other methods in terms of SSIM and PSNR.

In an application to imaging prostate, Hong et al. presented a Bayes shrinkage-based fused wavelet transform (BSbFWT) and Block-based autoencoder network (BBAuto-Net) for removal of noise from prostate MR images [121]. The method was tested on prostate mp-MRI data obtained from 1.5-T general electric (GE) and 3.0-T Siemens scanners with promising results obtained in comparison with conventional filters such as anisotropic, bilateral, Gabor, Gaussian, mean, NLM, wavelet, Wiener, autoencoders, and autoencoders with NLM filters. Li et al. [122] carried out a study on clinical abdominal MR images where their proposed method was based on a cascaded multi-supervision convolutional neural network named CMSNet. CMSNet showed superior noise reduction capabilities not only on Rician noise in MR images but also on low-dose perfusion noise in CT images. Juneja et al. [123] utilized dDLR in order to assess the image quality of conventional respiratory-triggered 3D magnetic resonance cholangiopancreatography (Resp-MRCP) and breath-hold 3D MRCP (BH-MRCP). Their experiments were done is 1.5-T setting using 42 patients, and two radiologists rated the visibility of the proximal common bile duct (CBD), pancreaticobiliary junction, distal main pancreatic duct, cystic duct, and right and left hepatic ducts in the final images. The main conclusion of this study indicated the feasibility of dDLR for BH-MRCP and Resp-MRCP.

Low-field MRI, in particular, has many benefits including affordability, compact footprint, and reduced shielding. SNR is linearly proportional to the main magnetic field (B0); hence, low-field MRI systems (< 1 T) inherently have significantly low SNR compared to conventional 1.5–3 T MRI scanners. Song et al. [124] proposed a CNN-based auto encoder network with a transfer learning approach to learn a data-driven transformation from high-field noisy data with application to 0.35-T pelvic MR images. Tajima et al. [125] studied the utility of a stacked U-Net method to reduce noise from the system. Their experiments on phantom as well as human MR images acquired on a 60–67mT MR scanner demonstrated improved qualitative and quantitative denoising performance. Table 3 summarizes the advantages and disadvantages of MRI denoising methods in the literature.

**Table 3:** Advantages and limitations for deep learning methods in MRI denoising

Literature	Main contribution	Advantages	Limitations
Kidoh et al. [55]	Proposed a novel denoising method (dDLR)	• Neuroradiologists’ assessments and experiments on a clinical dataset shows that dDLR outperforms other methods with respect to SSIM, PSNR	• Relatively small testing cohort • Experiments limited to T1, T2, FLAIR, and MPRAGE data
Zhu et al. [103]	Proposed a novel MR image denoising method called DESN which has a novel network architecture and a well-designed loss function	• A novel loss function which data fidelity loss, image quality penalty and three loss terms: MSE, SSIM, entropy term • Superior performance over SOTA in generating high-quality MR images with sufficient edge and texture information	• Requirement of a large amount of training image data pairs to learn the clear image prior information
Christopher et al. [105]	Proposed a variant of ADMM-DIP method for enhancing single coil MR images	• Achieved Rician noise removal from single MR image by utilizing the combined effect of MSE, KL divergence, and perceptual loss functions	• Experiments are done only on simulated data • Hyperparameter tuning is not optimized
Ran et al. [48]	Introduced a novel MRI denoising method based on the residual encoder–decoder Wasserstein GAN (RED-WGAN)	• Novel loss function combining perpetual loss from VGG-19 network with MSE and adversarial losses • Achieved superior performance over SOTA in simulated and clinical data • Comparatively better computational cost	• Experiments limited to brain data
Tian et al. [120]	Proposed a novel MRI image denoising method using the conditional GANs	• Experiments conducted on both synthetic and real clinical MRI datasets • Achieved high SSIM compared to SOTA in high noise levels	• Experiments are incomprehensive and performed on limited data
Chauhan et al. [14]	Proposed a combined approach of fuzzy logic and a convolutional autoencoder on a brain MR images	• The combined approach performs better than SOTA	• Experiments conducted on limited data
Jiang et al. [107]	Proposed the Multi-channel DnCNN (MCDnCNN) method with two main training strategies to denoise images with and without a specific noise level	• Comprehensive experiments conducted on public and clinical datasets • Reported high PSNR and SSIM over SOTA • Showed good generalizable applicability	• Model incompatibility with 3D volumetric data • Experiments confined to brain data
Tripathi et al. [108]	Propose a novel CNN-based denoiser called CNN-DMRI	• End-to-end training scheme utilizing residual learning scheme • Performance assessed qualitatively and quantitatively on simulated and real data • Capability to denoise without losing crucial image details	• Suboptimal computational time
Gregory et al. [109]	Proposed the HydraNet, a multi-branch deep neural network architecture that learns to denoise MR images at a multitude of noise level	• Compatible with numerous factors such as pulse sequences, reconstruction methods, coil configurations, and physiological activities	• Incompatible with volumetric denoising
Naseem et al. [111]	Proposed the Cross-Modality Guided Denoising Network (CMGDNet) for removing Rician noise in T1 data	**• **Compatibility with cross-modal medical imaging • Exploited complementary information existing in cross-modal images and improved the learning capability	• Experiments limited to public datasets • Experiments limited to brain data
Wu et al. [112]	Proposed a denoising method named 3D-Parallel-Rician Net, which combines global and local information to remove noise in MR images	• Introduced a powerful dilated convolution residual (DCR) module to expand the receptive field of the network • Introduced a depth wise separable convolution residual (DSCR) module to learn the channel and position information	• Evaluated only on simulated T1 MR image data • Requirement for high-quality noise-free ground-truth images
Singh et al. [113]	Proposed a noise filtering network which learns the image details from the image patches pixel-by-pixel from noise residuals to restore the detailed image features in an end-to-end feed-back approach	• Showed comparable performance with SOTA without losing important image information	• Insufficient number of experimentations
Tripathi et al. [115]	Proposed a dual path deep convolution network based on discriminative learning for denoising MR images	• Incorporated depth wise separable convolution to denoise the images of different noise levels • Yielded better performance as compared with various other networks • Attained favorable assessments from radiologists	• Experiments limited to public data and brain data
Yang et al. [118]	Proposed a hybrid regularization model from deep prior and low-rank prior. The local deep prior was explored by a fast flexible denoising convolutional neural network (FFDNet)	• Compared with the popular CS-MRI approaches, the experimental results demonstrated better performance	• Limited experiments
Moreno et al. [119]	Evaluated two unsupervised approaches for MRI denoising in the complex image space using k-space data: SURE and blind spot network	• Methods are evaluated on real knee MRI and synthetic brain MRI data • Both networks outperformed NLM and prove to be dependable denoising methods	• Experiments limited to public datasets • Incomprehensive experiments
Panda et al. [28]	Utilized perceptual loss and MSE for training a network for brain MRI denoising	• Restored images were visually desirable and contained more anatomically refined features • The proposed CNN network surpassed SOTA for Rician MRI denoising and obtained high quality brain MR images	• Comparatively large computational cost for training • Experiments on limited datasets

### Noise in Functional, Perfusion-Weighted, Diffusion-Weighted, and Flow MRI

Functional MRI (fMRI) is a prominent imaging technique for functional brain mapping and identification of functional networks. fMRI data is normally acquired using a fast EPI readout with T2*-weighted contrast to capture blood oxygenation level-dependent (BOLD) signals. Both spatial and temporal noise can hinder accurate identification of functional brain maps and function networks. Yang and colleagues applied a time-dependent deep neural network (DeNN) to denoise fMRI time series in individual brain regions [31]. The authors compared DeNN with several nuisance noise regression methods and validated the method using the Alzheimer’s Disease Neuroimaging Initiative (ADNI) database. DeNN identified unbiased correlations between a seed region in the posterior cingulate cortex and the default mode network and task-positive networks. The DeNN whole brain functional connectivity maps were three times as homogeneous as the functional connectivity maps obtained from the raw datasets. Zhao et al. introduced a data-driven deep learning approach based on a 3D convolutional long short-term memory (LSTM) network (3DConv-LSTM) and an adversarial network to generate noise-free realistic fMRI volumes [126]. They tested the method on both task and resting-state fMRI (rs-fMRI) data, compared it with state-of-the-art alternative methods, and concluded that for HCP and ABIDE datasets, the approach performed comparatively better using PSNR, SSIM, and MSE metrics. Le et al. [127] proposed a framework to detect noise components for rs-fMRI which involved several CNN models that jointly learnt spatial and temporal features using a majority voting strategy that constituted a faster noise detection process for rs-fMRI. Kam et al. [128] proposed a CNN framework for automatic rs-fMRI denoising which simultaneously learns spatio-temporal features of noise. Their studies further depicted visual explanations on how CNNs behave in the presence of noise in rs-fMRI, and the proposed framework illustrated high performance on multiple datasets including infant cohorts.

Compared with anatomical MRI, diffusion-weighted imaging suffers from low signal to noise ratio due to the application of diffusion gradients and fast EPI data readout. CNNs and deep image prior-based denoising methods have been developed in order to improve SNR in diffusion MRI. Lin et al. used a deep image prior (DIP) to simultaneously denoise all diffusion-weighted images. The method demonstrated superior performance when compared with the local principal component analysis method using both simulated and in vivo datasets [129]. Kawamura et al. evaluated the application of CNN-based denoising for multi-shot EPI DWI and compared the deep denoiser with other methods including block-matching and 3D filtering [130]. Zormpas-Petridis et al. developed a model to improve the image quality of whole-body diffusion-weighted imaging [24]. The study was conducted on both retrospective and prospective patient cohorts to optimize a denoising image filter (DNIF) deep learning model. Kaye and colleagues investigated the feasibility of accelerating prostate diffusion-weighted imaging using a novel guided denoising convolutional neural network (guided DnCNN) [32]. They carried out experiments on prostate DWI scans gathered from six single-vendor MRI scanners and produced images guided by the DnCNN with improved PSNR in comparison to the original DnCNN. The conventional deep learning techniques often require additional high-SNR data for supervising training. However, Tian et al. developed a self-supervised deep learning-based method referred to as “SDnDTI” for denoising diffusion tensor MRI data [131], which does not require additional high-SNR data, and the experiments carried out on DWI volumes provided by the Human Connectome Project (HCP) illustrated results with image sharpness and textural details.

Perfusion-weighted MRI images acquired using the arterial spin labelling (ASL) protocols are also based on an EPI sequence and suffer from low SNR. Xie et al. applied CNNs with dilated convolution kernels and wide activation residual blocks to preserve image resolution while suppressing noise [132]. The results suggested potentially 75% faster ASL acquisition without sacrificing accuracy in the estimation of cerebral blood flow. An unsupervised network to improve the SNR in ASL images [13] used each subject’s corresponding T1-weighted image as input to the network with noisy ASL images as labels. Hales et al. evaluated the performance of a denoising autoencoder (DAE) [15] for denoising ALS datasets. The work used 131 pediatric neuro-oncology patients to train the network and test the model performance for eleven healthy adult subjects. They compared the autoencoder with both Gaussian and non-local filters and reported a 62% SNR increase in the raw ASL images. The DAE denoised images demonstrated best fit to the Buxton kinetic model with a 75% reduction in the fit errors in comparison with the raw images. Several other recent works have proposed deep learning solutions for ASL MRI denoising [133–137].

MRI flow measurements are vulnerable to acquisition noise, velocity aliasing, and phase offset artefacts in clinical applications. These complications represent significant challenges for the analysis of small vascular structures including identification of intracranial aneurysms and treatment for near-wall regions. Several studies have attempted to use deep learning to reduce noise in flow MRI. Sun and co-workers proposed a physics-constrained deep learning approach that effectively reduced the measurement noise [138]. The method was verified using multiple test cases with synthetic vascular flow data. Similar studies were conducted by Fathi and colleagues who proposed a purely data-driven method to denoise 4D-flow MRI data [139].

## Image Resolution Enhancement

Spatial resolution is a key data acquisition parameter that impacts diagnostic accuracy and decision of subsequent clinical workflows. However, scans with higher spatial resolution often leads to longer data acquisition time and poorer signal to noise ratio and can be prone to motion artefacts. Post-processing resolution enhancement algorithms, such as zero-padding to increase the matrix size of the final image, have been widely applied in medical imaging. Further, methods such as B-splines and cubic interpolation can provide image resolution improvements without the use of prior models. Van Reeth and colleagues reviewed a general forward model for MR image resolution including geometric transformation, instrument point spread function, and downsampling during data acquisition [140] as well as conventional image super-resolution algorithms to solve the forward model, including iterative back-projection and regularization methods using priors.

For the past decade, deep learning-based image resolution enhancement has been widely adopted in the computer vision literature [141]. Dong et al. provided a detailed survey of deep learning methods for image super-resolution applications in computer vision and noted limitations in real-world scenarios [142]. These advances have inspired deep learning based super-resolution methods in MRI applications with promising results.

Partial volume effect (PVE) is key consideration in MRI super resolution, especially in volumetric and multi-slice MRI acquisitions where multiple tissue types are present in a single voxel. In such a scenario, the intensity of the resultant image depends on the collective contribution of each tissue. As a result, with high section thickness (usually 2.5 to 4 mm) and slice gaps, the risk of missing subtle anatomical features and lesions is high. To overcome this, Chaudhari et al. [164] proposed DeepResolve, a deep learning-based solution to resolve high-resolution thin slices from considerably thicker slices. The authors compared their deep learning solution with other through-plane interpolation techniques such as tricubic interpolation, Fourier interpolation, and sparse-coding super-resolution; however, DeepResolve illustrated significant superiority over other methods in terms of structural SSIM, PSNR, and RMS error scores.

### Spatial Resolution in Anatomical MRI

Anatomical MR scans with high in-plane resolution and minimal through-plane resolution can successfully reduce imaging time and improve image SNR. However, the resultant images have poor through-plane resolution and large partial volume errors. To address this problem, Zhao et al. exploited a deep learning approach called Synthetic Multi-Orientation Resolution Enhancement (SMORE) [143] for both anti-aliasing and super-resolution imaging. The method consists of a self-supervised anti-aliasing deep network followed by a super-resolution deep network, with application along different orientations within an image. SMORE demonstrated improvement in visualization and quantification for both brain and cardiac imaging applications. While most deep learning-based MR super-resolution methods report experiments using brain data, there is a considerable literature also focused on cardiac, fetal, and knee imaging applications.

#### Brain

The brain has fine anatomical structures and often suffers from partial volume errors from low resolution data acquisitions. Compared with conventional MRI resolution enhancement methods, deep learning models have shown superior performance.

Pham and colleagues studied multiscale trained 3D networks with knowledge transferred from different acquisition protocols to improve spatial resolution of brain images [144]. Chen et al. proposed a lightweight CNN model which operates on 3D patches of single channel inputs [145], and Sui et al. introduced a novel gradient-guided super-resolution method for enhancing isotropic images from anisotropic acquisitions [42]. Recently, Xue et al. developed a progressive sub-band residual learning super-resolution network (PSR-SRN) [146]. Li et al. used 305 paired brain MR images to train a two-step learning architecture called DeepVolume that combined CNNs with RNNs [45]. The two-step architecture consisted of a brain structure-aware network, in which the axial and sagittal MR images were fused by a multitask 3D U-net, and a spatial connection-aware network in which the resolution of the image was further enhanced by a LSTM block on a 2D (slice by slice) basis. The incorporation of RNNs enabled the DeepVolume architecture to achieve state of the art results in MRI super resolution.

Using residual learning, Shi and colleagues developed two algorithms for brain MR images. Their multi-scale residual learning network for image super resolution combined both multi-scale global residual learning (GRL) and shallow network block-based local residual learning (LRL) [147]. The LRL module effectively captured high-frequency details by learning local residuals, while the conventional GRL module enabled learning of high-resolution image details. They proposed a progressive wide residual network with a fixed skip connection (named FSCWRN) to combine global residual learning and shallow network-based local residual learning [148] and reported superior performance compared to the SRCNN [141], SRF [149], and VDSR [150].

By leveraging high-resolution images from 7 T MRI, Zhang et al. developed a parameter-efficient butterfly network that employed a dual spatial and frequency domain DL model for mapping between 3 and 7 T image pairs that produced improved image resolution at 3 T [151]. The results demonstrated superior performance over conventional methods both qualitatively and quantitatively. Several other groups have also shown promising results using deep learning models for brain MRI [152, 153].

Since their introduction [154], GANs have been utilized in many applications related to natural image processing as well as medical imaging applications including super-resolution MRI [152, 155–157]. The application of GANs in super-resolution MRI has generated high-resolution output images that are barely distinguishable from the original high-resolution images. Lyu et al. introduced the GAN-CIRCLE (constrained by the identical, residual, cycle learning ensemble) which realized super resolution in both MRI and CT [158]. The model achieved two-fold resolution improvement on brain images. Chen et al. introduced a 3D neural network design with a multi-level densely connected super-resolution network (mDCSRN) with generative adversarial network (GAN)-guided training [159]. The mDCSRN architecture outperformed other deep learning methods with four times higher resolution for T1-weighted images in a sixth of the computational time.

#### Other Anatomical Regions

Localization of pathology is extremely challenging when data acquisition is constrained due to physiological motion and other practical limitations. Cardiac MRI is often acquired with a small data matrix that results in low spatial resolution. Masutani and co-workers evaluated the application of a CNN to enhance anatomical detail in comparison with zero padding and bicubic interpolation methods. In an evaluation of 400 MRI scans, the DL model significantly outperformed zero padding and bicubic interpolation methods in 99.2% of the image slices [160]. Mahapatra and colleagues developed a method using progressive adversarial networks (P-GANs) to improve image resolution [161]. They used a triplet loss function to optimize their model, and the results on cardiac and retinal images demonstrated improved quality.

**Fig. 6:**
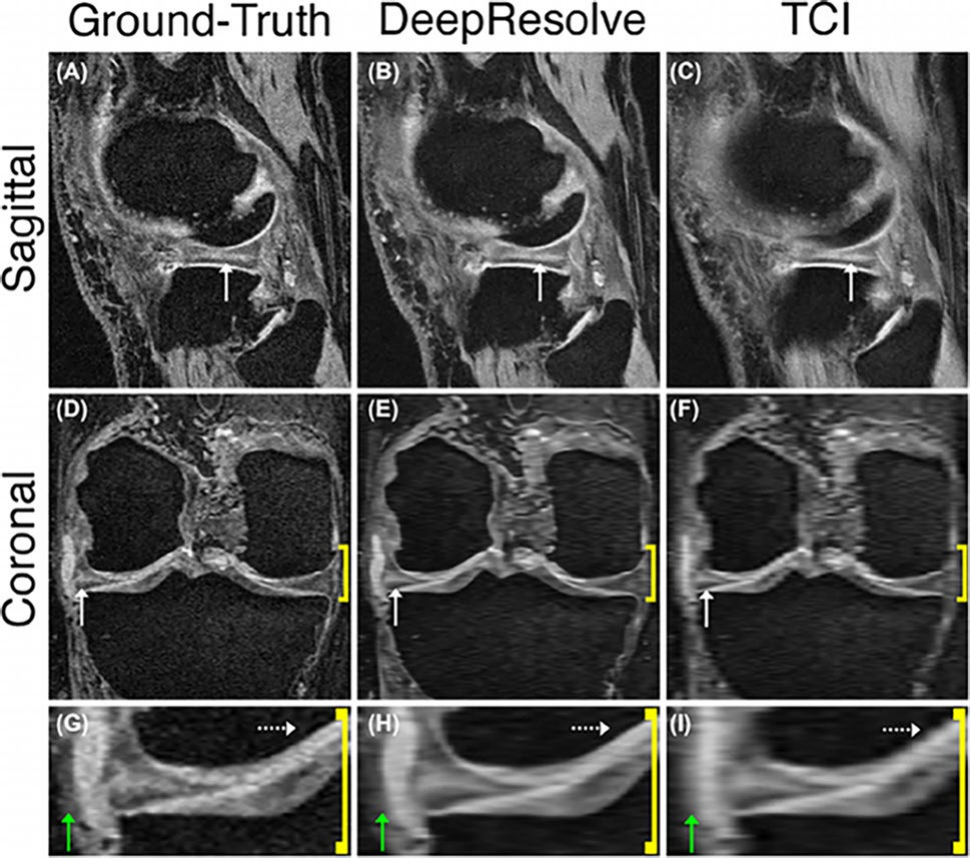
Example of a horizontal tear in the body of the lateral meniscus can be identified with the hyperintense double echo in steady-state signal. First column, high-resolution ground-truth; second column, DeepResolve; and third column, tricubic interpolation (TCI). Compared with the Ground-Truth, the DeepResolve image shows considerably less blurring to TCI images [163]

The low resolution in knee MR scans adversely affects the diagnosis of conditions such as knee osteoarthritis. To address this problem, Qiu et al. [162] used an efficient medical image super-resolution (EMISR) method by combining SRCNN with an efficient sub-pixel CNN (ESPCN). The authors demonstrated that EMISR outperformed both SRCNN and ESPCN alone using knee images from the IDI dataset, with the resultant images from the method having clearer anatomical boundary details. Chaudhari et al. applied a 3D CNN called DeepResolve to learn a model between low-resolution and high-resolution image. The trained model was applied to 17 knee patient datasets and compared with existing methods including clinically utilized tricubic interpolation (TCI), Fourier interpolation (FI), as well as the single image sparse-coding super-resolution (ScSR) method (Fig. 6). The results demonstrated the superior performance of DeepResolve with respect to SSIM, PSNR, and root-mean-square-errors (RMSE) as well as radiology assessment [163]. In a following study, Chaudhari and co-workers quantitatively demonstrated that super resolution minimally affects perceived global image blur and qualitatively that it minimally biases cartilage and osteophyte biomarkers and image quality. The study concluded that super resolution is more effective than naïve interpolation for accelerated image acquisition [43] (Fig. 6).

Fetal MR images are generally acquired with low resolution to avoid motion artefacts. McDonagh et al. applied a context-sensitive super-resolution method on 145 fetal MRI scans [164]. The model semantically adopted to input data by learning organ-specific features and generated high-resolution images with sharp edges and fine details that yielded an increased PSNR of 1.73 dB when applied on motion corrupted fetal data. McDonagh et al. [165] proposed a self-supervised super-resolution framework dynamic fetal MRI where low- and high-resolution samples are taken from simulated interleaved acquisitions. This framework also considers temporal information of the scan data during the self-supervised training process and is able to improve image quality and recover more image visual details.

Park et al. [21] introduced an autoencoder-inspired convolutional network super-resolution (ACNS) method which extrapolated missing spatial information using a nonlinear mapping between low-resolution and high-resolution features. The experiments were carried out on virtual phantom images and thoracic MRIs from four volunteers. The ACNS method produced results comparable with popular SRCNN, FSRCNN, and DRCN methods but with comparatively shorter computational times enabling real-time resolution enhancement of 4D imaging in MRI-guided radiation therapy.

Imaging the abdomen is important for many oncology applications such as prostate cancer. Thus, SR in prostate MR can facilitate early diagnosis and thereby influence the commencement of early treatments. Xu et al. [166] proposed an SR framework based on MSG-GAN and CapsGAN to produce high-quality MR images. Their experiments were based on the PROSTATEx database, and they were able to achieve a PSNR of 19.77 for SR of 8X. Similarly, the works of Molahasani et al. [167] applied an SRGAN to enhance prostate MR images and improve the in-plane resolution by a factor of 8. Table 4 summarizes the advantages and disadvantages of MRI resolution enhancement methods in the literature.

**Table 4:** Advantages and limitations for deep learning methods in MRI resolution enhancement

Literature	Main contribution	Advantages	Limitations
Zhao et al. [144]	Reviewed the SMORE algorithm and demonstrated its potential use in both research and clinical scenario	• SMORE showed improved visualization of brain white matter lesions in FLAIR images acquired from multiple sclerosis patient • Showed improved visualization of scarring in cardiac left ventricular remodeling after myocardial infarction • Showed improved performance in parcellation of the brain ventricular system • Both visual and quantitative metrics of resolution enhancement are demonstrated	• Limited methodology contribution; based on a previously introduced SR technique called SMORE
Pham et al. [145]	Studied deep 3D CNNs for the super-resolution of brain MRI	• Investigated monomodal super-resolution in terms several factors: optimization methods, weight initialization, network depth, residual learning, filter size in convolution layers, number of the filters, training patch size, and number of training subjects • Emphasized that one single network can efficiently manage multiple arbitrary scaling factors based on a multiscale training approach • Extend super-resolution networks to the multimodal super-resolution using intramodality priors • Investigate the impact of transfer learning skills onto super-resolution performance in terms of generalization among different datasets • Learnt models were used to enhance real clinical low-resolution images	
Chen et al. [146]	Introduced a novel 3D Densely Connected Super-Resolution Networks (DCSRN) to restore HR features of structural brain MR images	• Experiments conducted on clinical data demonstrated superior performance over bicubic interpolation as well as other deep learning SOTA in restoring 4 × resolution-reduced images	• Insufficient experiments and datasets utilized in the study
Sui et al. [42]	Developed a model to construct images with spatial resolution higher than can be practically obtained by direct Fourier encoding	• Provided an estimate of the spatial gradient prior from the LR inputs for the HR reconstruction • Incorporated the anisotropic acquisition schemes • The model was trained over the LR images themselves only • Developed a closed-form solution to the SRR model was developed to obtain the HR reconstruction • Assessed performance on the simulated and clinical data • Reported superior SRR over SOTA • Obtained better images at lower or the same cost in scan time than direct HR acquisition	• Experiments limited to brain data
Xue et al. [147]	Proposed the progressive sub-band residual learning SR network (PSR-SRN) which contained focused on missed high-frequency residuals as well as on reconstructing refined MR image	• Introduced a brain-like mechanisms (in-depth supervision and local feedback mechanism) and progressive sub-band learning strategy to emphasize variant textures of MRI • Illustrated superior performance over traditional and deep learning MRI SR methods	• Experiments only on brain data
Li et al. [45]	Introduced DeepVolume, a two-step deep learning architecture to address the challenge of accurate thin-section MR image reconstruction	• Extensive experiments illustrate that DeepVolume can produce SOTA reconstruction results by embedding more anatomical knowledge • Practical and clinical value of DeepVolume is validated by applying the brain volume estimation and voxel-based morphometry • Reliable brain volume estimation in the normalized space based on the thick-section MR images compared with SOTA	• Experiments may be not dependable when SPM cannot provide accurate segmentation results
Shi et al. [148]	Proposed a novel residual learning-based SR algorithm for MRI, which combines multi-scale GRL and shallow network block-based LRL	• Simulated and real MRI datasets used for evaluation • Superior performance over SOTA CNN-based SR algorithms	• High computational complexity • Incompatible with 3D data
Shi et al. [149]	Proposed a progressive wide residual network with a fixed skip connection (FSCWRN)	• Ability to relax the problems of feature degradation and diminishing feature reuse • Experiments on real and simulated data show competitive performance over SOTA	• Incompatible with 3D data
Kang et al. [153]	Presented a novel HR-MRI generation approach based on CNNs and multi-resolution analysis	• Improved resolution T2 data with the prior information provided by HR T1 data • Took advantage of structural similarity between the modalities • Experiments on a real MRI dataset show improved over SOTA single- and multi-modal networks • Effectively restore the edge details even with 4 × magnification	• Experiments only on brain data
Dong et al. [154]	Presented MAU-Net for spatial resolution enhancement, based on the observation that multi-parametric MRI provide relevant priors for MRSI enhancement	• Model trained on in vivo brain imaging data from patients with high-grade gliomas • Combined loss function consisting of pixel, structural, and adversarial loss • Ability to reconstruct high-quality metabolic maps with a high-resolution of 64 × 64 from a low-resolution of 16 × 16 with better performance over SOTA	• Limited experiments and datasets utilized
Zhang et al. [156]	Proposed SOUP-GAN: Super-resolution Optimized Using Perceptual-tuned Generative Adversarial Network in order to produce thinner slices	• Outperformed other conventional resolution-enhancement methods and previous SR work on medical images based on both qualitative and quantitative comparisons • A novel 3D SR interpolation technique, providing potential applications for both clinical and research applications	
Sui et al. [157]	Designed a deep architecture that utilizes an adversarial scheme with a generative neural network against its degradation counterparts	• Achieved high-quality brain MRI at an isotropic resolution of 0.125 mm^3^ with 6 min of imaging time • Experiments on simulated and clinical data • Demonstrated superior SRR results SOTA deconvolution-based methods	• Limited experiments and datasets
Jiang et al. [158]	Proposed the Fused Attentive Generative Adversarial Networks(FA-GAN) to generate SR MR image from LR images	• Designed a global feature fusion module, including the channel attention module, the self-attention module, and the fusion operation in order to enhance the key features MR images • Introduced the spectral normalization process to make the discriminator network stable during training	• Relatively small training cohort • Insufficient experiments and datasets
Chen et al. [160]	Proposed a novel 3D neural network design, namely, a multi-level densely connected super-resolution network (mDCSRN) with GAN-guided training	• Trains and infers quickly relative to other SOTA • Promotes realistic output • Experiments on a real MR data show superior performance recovering 4 × resolution-downgraded images and runs 6 × faster compared with SOTA	• Limited experiments and datasets

### Resolution in Diffusion-Weighted, Flow, and Spectroscopic MRI

While diffusion-weighted MRI allows high-resolution imaging at high magnetic fields and high gradient strength for long scan times, the image spatial resolution and number of diffusion directions are restricted. Several deep learning models have been developed to enhance the quality of diffusion images. Qin et al. developed a super-resolution q-space deep learning method [46] to estimate high-resolution tissue microstructure based on under-sampled q-space signals. The work extended the earlier q-space DL methods [168, 169] that used super-resolution models. The authors evaluated the methods using the two Human Connectome Project datasets [170, 171] to produce accurate tissue parameters. Albay et al. presented a novel GAN-based deep neural network model to obtain high-resolution images from low-resolution diffusion images [172]. The work provided a proof of principle for the effectiveness of GAN to increase the spatial resolution by twofold for diffusion MRI. Chatterjee et al. [173] introduced the ShuffleUNet, a single image SR technique which involved pixel shuffle operations for improved down- and up-sampling capabilities. Their experiments on the IXI dataset achieved very high SSIM values such up to 0.913.

Fathi and co-workers developed a DL approach for super resolution and denoising of 4D-flow MRI. They used flow physics to regularize a DNN model to improve its convergence properties with limited training data [139]. Flow velocities, pressure, and the MR image magnitude were modelled as a patient-specific DNN. Experiments on numerical phantoms demonstrated increased spatial resolution by a factor of 100 and increased temporal resolution by a factor of 5 in comparison to simulated 4D-Flow MRI.

MR spectroscopy (MRS) aims to quantify tissue metabolism. However, due to the low concentration of most metabolites, their identification in proton MR spectra is a difficult task. Iqbal et al. [174] proposed a densely connected UNet (D-UNet) architecture capable of producing super-resolution MRS images. The model was trained using inputs from both T1-weighted images and low-resolution MRS images, and the labelled output super-resolution MRS images were simulated by combination with segmented white matter and gray matter images [160].

## Challenges and Future Perspectives

Deep learning models have shown promising results for the reduction of image artefacts and noise and the improvement of image resolution. Compared with conventional machine learning methods, deep learning models show consistently better performance in these post-processing tasks. Furthermore, deep learning methods are also computationally efficient during inference in comparison with many conventional iterative algorithms. Significantly, the promising initial results have motivated imaging device manufacturers to increase the range of deep learning-based solutions in their product portfolios [175]. However, while there is an overall consensus that deep learning methods are playing a critical role in the future of medical imaging, there are a number of major challenges yet to be addressed.

### Data Availability and Open Datasets

Most deep learning models require a large amount of training data to avoid model over-fitting. However, good quality training sets of medical images in general are difficult to obtain due to privacy or other availability issues. Furthermore, MR image quality and contrasts are varied and highly dependent on anatomical regions, sequence parameters, and hardware configurations [176]. These factors make it challenging to collect a large cohort of good quality datasets with sufficiently diverse MRI contrast parameters and anatomical regions. The lack of training data can lead to significant bias in the performance of deep learning models [177].

Existing open MRI datasets that are available in the literature can add significant value in the development of deep learning methods. Example datasets include the IXI dataset (brain-development.org/ixi-dataset), fastMRI [178], mridata (mridata.org), AOMIC [179], OCMR (registry.opendata.aws/ocmr_data), HCP (humanconnectomeproject.org), and the UK Biobank (ukbiobank.ac.uk). There are a number of open datasets hosted in OpenNeuro such as the Monash fMRI-fPET data [180], and disease-specific datasets including ADNI (adni.loni.usc.edu), and the ENIGMA study data (enigma.ini.usc.edu). Bento et al. [181] have provided a comprehensive review of multi-site structural brain MRI datasets.

Simulated datasets can help to mitigate the lack of in vivo training datasets. For example, simulated head motion datasets can be used to train a network for application in clinical datasets with head motion [19, 182]. During the evolution of MRI technology, physics-based MRI simulators have been an active research area that has added significant value to the development of deep learning models. For example, Xanthis and colleagues developed a GPU-based realistic motion simulation [183], JEMRIS is a widely used MRI simulator [184], and the FSL toolkit includes the POSSUM MRI simulator [185].

### Generalizability

Out-of-distribution (OOD) data refers to inputs that are drawn from a distribution different to that of the training dataset. OOD data is to be expected in medical imaging applications of deep learning models due to limited training datasets, scanning protocol variation, and the potential for an image to include uncommon or rare disease features. The performance of a deep learning model to OOD input is an important consideration for the assessment of its generalizability. To date, there is only a limited literature that assesses the reliability of deep learning models on clinical OOD data [186].

Modelling data and parameter uncertainties can provide significant insight and assess risks for dealing with unseen datasets. Tanno and colleagues investigated uncertainty modelling for diffusion MRI super-resolution and sought to provide a high-level explanation of deep learning models with respect to variation in input datasets [187]. A number of similar works applied explicit uncertainty models during model training and inference in order to assess model robustness and uncertainties associated with input data [46, 188, 189]. Hallucinations are false image features introduced when an imperfect or inaccurate model prior is used during image processing and typically occur when training and testing have different data distributions. Hallucinations heavily related to the stability of a model especially when modelling an inverse problem. An estimate of hallucinations is of significant interest in image reconstruction as well as image super-resolution.

Unsupervised training using unpaired datasets can learn to disentangle data and artefact representations in a latent space [190] to improve model generalizability to new datasets. Deep image prior is a self-learning technique for regularized inverse problems without the need to pre-train the model [191] that successfully demonstrated greater generalizability to new inputs.

### Clinical Validation

An important observation from the current literature is that most current deep learning methods have only been demonstrated in a small cohort of datasets at a single imaging site. A thorough evaluation of AI algorithms is essential before their full clinical utilization. In an opinion paper, Kelly and colleagues argued that clinical validation of artificial intelligence should be carefully considered in different test scenarios to gain insight for potential biases and variations [192]. Furthermore, the current medical device regulatory processes are not designed for continuous evaluation of AI algorithms capable of transfer learning and domain adaptation. Improvement in these guidelines can provide clearer pathways to enable the clinical utility of deep learning models.

Prospective and multi-site evaluation of deep learning models is important to identify unknown variations from datasets and models applied in real-world environments. In a prospective study, Rudie and colleagues have evaluated DL-based brain MRI enhancement for increase in SNR, anatomic conspicuity, overall image quality, imaging artefacts, and diagnostic confidence, assessed by four board-certified neuroradiologists [193]. Similarly, Bash et al. [194] have conducted prospective evaluation of DL-based denoising using five scanners across five sites in 61 patients undergoing spinal MRI scans. They have compared standard clinical care images with images obtained from DL enhancement. In [195], Chaudhari et al. have outlined the challenges for prospective deployment of MRI in clinical practice and emphasized the importance for reproducibility of research studies through the sharing of datasets and software. Currently, most of validation studies are still performed with carefully defined clinical protocols using a limited number of subjects. We anticipate that increasingly more studies will be performed due to the importance of validation of AI in the real world.

The drive to develop explainable deep learning models is another important step for building trust in AI algorithms. The instability issue demonstrated during imaging and other potential bias during development are key aspects to address during clinical validation. Domain knowledge can be incorporated into deep learning models to improve their overall performance and reliability [196]. Specifically, for MRI, domain knowledge can be derived from either physics knowledge of MRI instruments, physiological information from patient studies, and from general clinical knowledge. Domain knowledge not only provides a way to augment deep learning models to improve model performance, but also offers guidance to deep learning models when dealing with uncertain scenarios during clinical evaluation and utilization.

## Conclusion

In this review paper, we have provided an overview of deep learning methods for post-processing MR images including reduction of image artefacts, suppression of image noise, and enhancement of image resolution. Throughout the literature, there is consistent evidence that improved image quality can be achieved using deep learning methods in comparison with conventional techniques. However, the current challenges and future perspectives for data availability, generalizability, and clinical validation of deep learning algorithms highlight the requirement for a concerted and ongoing research effort in this rapidly evolving discipline.
